# Transformed Waldenström Macroglobulinemia Responsive to Tafasitamab Plus Lenalidomide: A Case Report

**DOI:** 10.7759/cureus.32403

**Published:** 2022-12-11

**Authors:** Syed Alishan Nasir, Deep Pandya, Steven Wojkiewicz, Bhavna Khandpur, Elizabeth Downes, Pradip Pathare, Richard Frank

**Affiliations:** 1 Internal Medicine, Norwalk Hospital, Norwalk, USA; 2 Lead Bioinformatics, Ruby L. Ruggles Biomedical Research Institute, Nuvance Health, Danbury, USA; 3 Radiology, Norwalk Hospital, Nuvance Health, Norwalk, USA; 4 Pathology, Norwalk Hospital, Nuvance Health, Norwalk, USA; 5 Hematology and Medical Oncology, Physician Assistant Program, Sacred Heart University, Fairfield, USA; 6 Radiation Oncology, Norwalk Hospital, Nuvance Health, Norwalk, USA; 7 Hematology and Medical Oncology, Ruby L. Ruggles Biomedical Research Institute, Nuvance Health, Norwalk, USA

**Keywords:** high grade lymphoma, tafasitamab & lenalidomide, histology transformation, waldenstrom macroglobinaemia, diffuse large b cell lymphoma (dlbcl)

## Abstract

The histologic transformation (HT) of Waldenström macroglobulinemia (WM) into diffuse large-cell lymphoma is an uncommon but poor-prognostic event for which there is no standard therapy. Knowledge of this entity is mainly derived from largely retrospective studies, which report abysmal average survival rates even with the utilization of first-line chemoimmunotherapy and especially in patients who meet the high-risk criteria based on prognostic indices used for WM.

We present the case of a 75-year-old man with high-risk, transformed WM who was ineligible for standard chemoimmunotherapy (due to pancytopenia and multiple comorbidities) and was consequently treated with tafasitabmab, an anti-CD19 monoclonal antibody plus lenalidomide. Tafasitamab plus lenalidomide (TAF/LEN) is a recently approved therapy for relapsed or refractory de novo diffuse large-cell lymphoma (DLCL) but has not been previously studied in transformed low-grade lymphomas or WM. We show that TAF/LEN resulted in a complete and durable response of the DLCL by PET/CT and a complete bone marrow response of lymphoplasmacytoid cells, including the normalization of complex cytogenetic abnormalities.

The extraordinary response of our patient to TAF/LEN suggests that this combination may be an effective and tolerable therapy for transformed WM as well as relapsed or refractory non-transformed WM. Clinical trials of TAF/LN for the treatment of Waldenström macroglobulinemia are recommended.

## Introduction

The treatment landscape for Waldenström's macroglobulinemia (WM) has greatly expanded over the past decade with the introduction of novel alkylating agents, Bruton’s tyrosine kinase inhibitors, and proteasome inhibitors, all typically combined with the anti-CD20 monoclonal antibody rituximab [1]. While these novel agents have greatly improved the prognosis of classic WM, they are ineffective for WM that has undergone histologic transformation (HT) into diffuse large cell lymphoma (DLCL). HT is an uncommon event associated with a poor prognosis despite the use of chemoimmunotherapy [2,3]. Moreover, many patients with HT are ineligible for aggressive chemotherapy due to age, comorbidities, and a reduced bone marrow reserve. New treatments for HT are, therefore, urgently needed.

We present the case of an older WM patient who experienced HT in the setting of prior therapies, a low-performance status, and profound cytopenias that precluded standard R-CHOP for DLCL. He experienced a complete and durable remission upon treatment with the anti-CD19 monoclonal antibody tafasitamab plus the immunomodulatory drug lenalidomide (TAF/LEN). This combination is only approved for use in non-transformed relapsed or refractory DLCL [4]. To the best of our knowledge, this is the first report of the use of TAF/LEN for HT or relapsed WM.

## Case presentation

An active 75-year-old man was first diagnosed with WM 19 years earlier, when he presented with an asymptomatic IgM kappa paraprotein of 1 gm/dL, a normal hemogram, and a bone marrow biopsy (BM) showing 30% infiltration by lymphoplasmacytoid cells consistent with lymphoplasmacytic lymphoma (LPL). Flow cytometry at the time showed (+)CD19/CD20/IgMk and (−)CD5/CD10/CD23, cytogenetics 46,X,-Y(3)/46,XY(17). He underwent watchful waiting for 10 years, after which he developed symptomatic grade 2 anemia with BM showing 95% infiltration with LPL; the IgM level at that time was 1083 mg/dL. He was treated with bortezomib, dexamethasone, and rituximab (R) for six months and remained well for three years until treatment was switched to bendamustine and rituximab (BR) for six cycles due to recurrent anemia. This resulted in improved symptoms and the partial response of IgM and BM infiltration by LPL. After completing BR, he developed disseminated herpes zoster complicated by foot drop and grade 2 peripheral neuropathy. He was observed for three years until he developed acute grade 4 cytopenias (WBC 0.7 × 109/L, hemoglobin 7 gm/dL, platelets <10 × 109/L), an IgM of 366 mg/dL, and a BM showing 90% LPL with MYD88L265P and CXCR4WHIM mutations. He received ibrutinib plus rituximab with normalization of blood counts and an IgM reduction to 84 mg/dL. After 20 months of ibrutinib, he developed atrial fibrillation with a left ventricular ejection fraction of 10-15%, which normalized with amiodarone. Ibrutinib was therefore discontinued.

**Figure 1:**
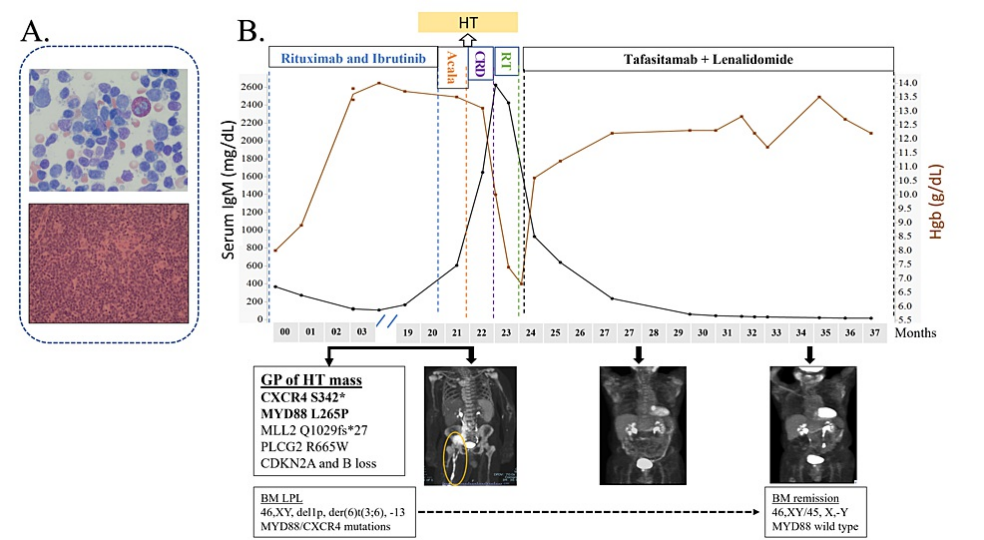
Clinical pattern of response to treatment of transformed Waldenström macroglobulinemia to tafasitamab and lenalidomide (A) Hematoxylin-eosin-stained section of the (top) bone marrow showing lymphoplasmacytic lymphoma and (bottom) lymph node showing diffuse large-cell lymphoma, taken at the time of HT. (B) Timeline of response of IgM (black line) and Hgb (brown line) as well as PET/CT images to tafasitamab and lenalidomide. The encircled region represents bulky lymphadenopathy and femur involvement by DLCL, which resolved with therapy. Cytogenetics and MYD88 mutation also improved with treatment (bottom boxes). BM: bone marrow, GP: genomic profile, HT: histologic transformation.

Acalabrutinib was briefly substituted; however, it was also not tolerated due to cytopenias. During this substitution, he developed a palpable pelvic mass causing painful lower extremity edema, grade 4 pancytopenia (WBC 1.2, Hgb 6 gm/dL, and platelets 25,000), an elevated LDH=331 U/L (ULN=222), and an IgM of 2,624 mg/dL. BM showed replacement by LPL (Figure 1A, top panel), cytogenetics 46,XY, del(1)(p22q34),der(6)t(3;6)(q12;q13),-13,+mar(2)/46,XY(1), MYD88L265P, and CXCR4WHIM mutations, but no TP53 mutation. PET/CT revealed metabolic activity throughout the visualized axial and appendicular skeleton, FDG-avid lymphadenopathy in the left supraclavicular and axillary regions, an enlarged spleen, numerous retroperitoneal nodes, with a 7.5 cm × 4.5 cm mass in the right hemipelvis (SUV 7.5 grams/mL) extending to the external iliac chain to a second large mass 10.5 cm × 4.8 cm/SUV 9.7, that surrounded the iliac vessels. The extranodal disease was present in a 3.0 cm × 2.6 cm right rectus femoris mass, the distal right femur, and a 7.3 cm × 4.7 cm right popliteal fossa mass (SUV 12.7). The latter was biopsied revealing DLCL, activated B-cell type (Figure 1A, bottom panel), (+)CD20/PAX5/CD21/BCL2/MUM1 and (-)BCL6/cyclin D1/CD23/CD10; FISH showed no BCL-2 or C-MYC translocations and the Ki-67 index was 80%. Genomic profiling with FoundationOne Heme (Foundation Medicine, Cambridge, MA) showed several alterations, including MYD88 and CXCR4 mutations confirming HT of WM (Figure 1B, bottom panel).

Our patient was ineligible for R-CHOP, the most commonly utilized regimen for HT (3), due to pancytopenia and multiple comorbidities. He was briefly treated with cyclophosphamide/rituximab/dexamethasone (CRD), which did improve thrombocytopenia and reduce the IgM (Figure 1B) but was complicated by urosepsis. In order to improve his abdominal pain and massive right leg edema, he received palliative radiotherapy (30 Gy in 12 fractions) for the bulky pelvic nodal mass. He then initiated tafasitamab (12 mg/kg) and dose-reduced lenalidomide (15 mg × 21/28 days) (4). He tolerated therapy well, with rapid improvement in his performance status, and after eight weeks of treatment, had normalization of hemoglobin and platelet counts, continued reduction in IgM, and a complete response by PET/CT (Figure 1B). The only grade 2 adverse effect was intermittent neutropenia, which required periodic filgrastim. After one year of therapy, PET/CT showed continued remission, and BM examination revealed a normocellular marrow with the absence of lymphoplasmacytoid cells by flow cytometry, normalization of cytogenetics, and a negative PCR for the MYD88 mutation. Lenalidomide was discontinued, and he remains on monthly tafasitamab maintenance therapy without clinical change at 18 months from the diagnosis of HT.

## Discussion

Histological transformation into DLCL is often a terminal event in WM, despite the use of chemoimmunotherapy [2,3,5]. A prognostic index predicting survival in HT has recently been published, categorizing patients into low-, intermediate-, and high-risk groups based on the presence of three criteria (elevated LDH, platelet count <100 × 109/L and prior treatment for WM). The high-risk disease is associated with a poor response to chemoimmunotherapy and a median survival of 4.8 months [5-7]. We present the case of a 75-year-old man with high-risk transformed WM who was ineligible for cytotoxic therapy but had a complete and durable response to the chemotherapy-free regimen of TAF/LEN. Moreover, this regimen was effective in treating both our patient’s lymph node-based DLCL as well as the LPL still present in the bone marrow, including a cytogenetic and molecular remission. Such a response could not have been predicted given the lack of development of lenalidomide plus rituximab for the treatment of WM [8].

**Figure 2:**
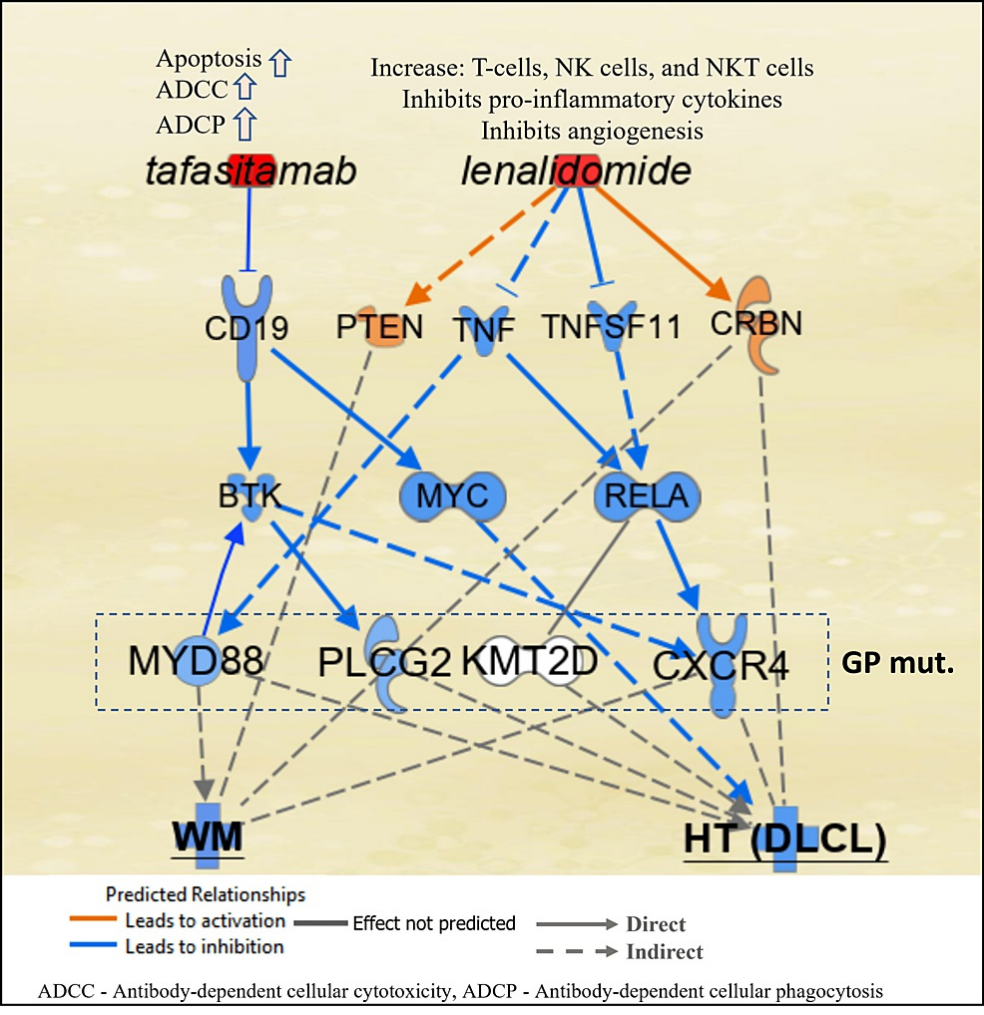
Schematic representation of the possible mechanism of action in B-cells of tafasitmab and lenalidomide against HT (DLCL) and WM Tafasitamab blocks CD19 signaling, resulting in inhibition of MYC and BTK. Lenalidomide activates CRBN and PTEN and inhibits tumor necrosis factor (TNF) superfamily members resulting in inhibition of MYD88 as well as CXCR4 through regulation of RELA (a subunit of NF-kB). The networks analyses were generated through the use of QIAGEN IPA [20]. GP mut=genomic profile mutations of the case presented. HT: histologic transformation, DLCL: diffuse large-cell lymphoma, WM: Waldenström's macroglobulinemia.

Tafasitamab is an Fc-enhanced, anti-CD19 antibody that is presently approved for use in relapsed/refractory DLCL in combination with lenalidomide [3]. CD19 is universally expressed in all cases of WM, making it a viable target for immunological therapies such as TAF [9]. The rationale for combining TAF/LEN is based on the stimulation and proliferation of NK cells by lenalidomide, coupled with the amplification of NK-cell-mediated, antibody-dependent cellular cytotoxicity by tafasitamab [10]. A simplified potential mechanism of action in B-cells for TAF/LEN is shown in Figure 2. TAF binding of CD19 inhibits MYC [11] and BTK [12-14], and LEN directly inhibits TNF superfamily members, resulting in the neutralization of gain-of-function mutations in CXCR4, MYD88, and PLCG2 [15-17]. LEN and TAF provide additional inhibition of these mediators and BCR and ILK signaling [18], as well as the tumor microenvironment [19], through effects on the cascade of proteins shown in Figure 2.

The treatment space for transformed lymphomas is in dire need of effective, non-toxic therapies that offer meaningful prolongations of life along with an acceptable quality of life. Included in this category are Richter’s transformation of chronic lymphocytic leukemia/small lymphocytic lymphoma and the diffuse-large cell transformation of indolent non-Hodgkin’s lymphomas, such as follicular lymphoma, marginal zone lymphoma, and lymphoplasmacytic lymphoma/WM [21,22]. Many patients experiencing large cell transformation after years of prior therapy for indolent lymphomas are ineligible for cytotoxic chemotherapy, allogeneic hematopoietic stem cell transplant, or chimeric antigen receptor T-cell therapy (CAR-T). The results presented herein suggest that tafasitamab plus lenalidomide might be a more tolerable and equally effective regimen for this patient population and should be tested in clinical trials specifically targeting the transformed lymphoma population.

## Conclusions

The chemotherapy-free regimen of tafasitamab plus lenalidomide may be an effective treatment for HT as well as non-transformed, relapsed WM. It is particularly well-suited to this population of patients characterized by older age, comorbidities, and reduced bone marrow reserve. Further investigation of TAF/LEN in HT/WM is warranted in clinical trials.
