# Correction: Transformed Waldenström Macroglobulinemia Responsive to Tafasitamab Plus Lenalidomide: A Case Report

**DOI:** 10.7759/cureus.c85

**Published:** 2022-12-14

**Authors:** Syed Alishan Nasir, Deep Pandya, Steven Wojkiewicz, Bhavna Khandpur, Elizabeth Downes, Pradip Pathare, Richard Frank

**Affiliations:** 1 Internal Medicine, Norwalk Hospital, Norwalk, USA; 2 Lead Bioinformatics, Ruby L. Ruggles Biomedical Research Institute, Nuvance Health, Danbury, USA; 3 Radiology, Norwalk Hospital, Nuvance Health, Norwalk, USA; 4 Pathology, Norwalk Hospital, Nuvance Health, Norwalk, USA; 5 Hematology and Medical Oncology, Physician Assistant Program, Sacred Heart University, Fairfield, USA; 6 Radiation Oncology, Norwalk Hospital, Nuvance Health, Norwalk, USA; 7 Hematology and Medical Oncology, Ruby L. Ruggles Biomedical Research Institute, Nuvance Health, Norwalk, USA

This article has been corrected to remove a duplicated figure and correct the corresponding figure mention. Cureus and the authors apologize that this was not identified prior to publication.

